# Assessing the validity of corneal power estimation using conventional keratometry for intraocular lens power calculation in eyes with Fuch’s dystrophy undergoing Descemet membrane endothelial keratoplasty

**DOI:** 10.1007/s00417-020-04998-w

**Published:** 2020-11-13

**Authors:** Raphael Diener, Maximilian Treder, Jost Lennart Lauermann, Nicole Eter, Maged Alnawaiseh

**Affiliations:** 1 Department of Ophthalmology, University of Muenster Medical Center, Albert-Schweitzer-Campus 1, Building D15, 48149 Muenster, Germany; 2 Department of Ophthalmology, Fulda Medical Center, Fulda, Germany

**Keywords:** Hyperopic shift, Conventional keratometry, DMEK, Triple DMEK, IOL power calculation, PA ratio, Posterior to anterior corneal curvature radii ratio

## Abstract

**Purpose:**

The present retrospective study was designed to test the hypothesis that the postoperative posterior to preoperative anterior corneal curvature radii (PPPA) ratio in eyes with Fuch’s dystrophy undergoing Descemet membrane endothelial keratoplasty (DMEK) is significantly different to the posterior to anterior corneal curvature radii (PA) ratio in virgin eyes and therefore renders conventional keratometry (K) and the corneal power derived by it invalid for intraocular lens (IOL) power calculation.

**Methods:**

Measurement of corneal parameters was performed using Scheimpflug imaging (Pentacam HR, Oculus, Germany). In 125 eyes with Fuch’s dystrophy undergoing DMEK, a fictitious keratometer index was calculated based on the PPPA ratio. The preoperative and postoperative keratometer indices and PA ratios were also determined. Results were compared to those obtained in a control group consisting of 125 eyes without corneal pathologies. Calculated mean ratios and keratometer indices were then used to convert the anterior corneal radius in each eye before DMEK to postoperative posterior and total corneal power. To assess the most appropriate ratio and keratometer index, predicted and measured powers were compared using Bland-Altman plots.

**Results:**

The PPPA ratio determined in eyes with Fuch’s dystrophy undergoing DMEK was significantly different (*P* < 0.001) to the PA ratio in eyes without corneal pathologies. Using the mean PA ratio (0.822) and keratometer index (1.3283), calculated with the control group data to convert the anterior corneal radius before DMEK to power, leads to a significant (*P* < 0.001) underestimation of postoperative posterior negative corneal power (mean difference (*∆* = − 0.14D ± 0.30) and overestimation of total corneal power (*∆* = − 0.45D ± 1.08). The lowest prediction errors were found using the geometric mean PPPA ratio (0.806) and corresponding keratometer index (1.3273) to predict the postoperative posterior (∆ = − 0.01 ± 0.30) and total corneal powers (∆ = − 0.32D ± 1.08).

**Conclusions:**

Corneal power estimation using conventional K for IOL power calculation is invalid in eyes with Fuch’s dystrophy undergoing DMEK. To avoid an overestimation of corneal power and minimize the risk of a postoperative hyperopic shift, conventional K for IOL power calculation should be adjusted in eyes with Fuch’s dystrophy undergoing cataract surgery combined with DMEK. The fictitious PPPA ratio and keratometer index may guide further IOL power calculation methods to achieve this.

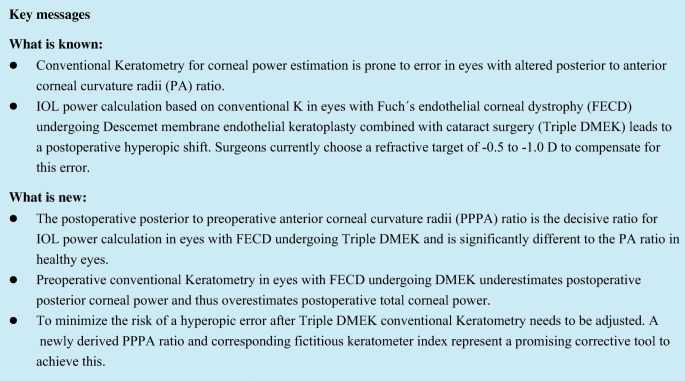



## Introduction

Intraocular lens (IOL) power calculation is traditionally based on keratometers that estimate the corneal refractive power from anterior corneal measurements, by using a standardized fictitious refractive index (1.3320) referring to a theoretical single refractive lens representing both corneal surfaces. This so-called conventional keratometry (K) is used in IOL Master PCI devices for corneal power estimation and assumes a constant posterior to anterior corneal curvature radii ratio (PA ratio or R_PA_) [1]. This assumption leads to sufficient refractive outcomes when IOL power calculation is performed in virgin eyes [2, 3].

However, when anterior corneal radius is altered by corneal refractive surgery, the PA ratio is disrupted and the usual keratometric refractive index becomes invalid. As a consequence, after myopic correction, conventional K readings usually overestimate corneal power and the resulting IOL power is underestimated, leading to postoperative hyperopia [4].

Similar, in eyes with Fuch’s dystrophy, a hyperopic shift has been reported when cataract surgery is combined with a Descemet membrane endothelial keratoplasty (triple DMEK) [5–13]. This has been attributed to regression of the posterior stroma edema associated with a steepening of the posterior corneal curvature [5–13]. However, as the posterior corneal curvature is not measured directly, neither a preoperative flat posterior corneal curvature, nor a postoperative change of posterior corneal radius is considered in the IOL power calculations based on measurement of the anterior corneal surface and conventional K [1]. Therefore, the decisive ratio for IOL power calculation in eyes with Fuch’s dystrophy, is the ratio between postoperative posterior corneal radius, once stable refraction is achieved, and preoperative anterior corneal radius, when conventional K is performed (Table 1).

**Table 1:** Demographic data

	Fuch’s dystrophy	Control
Subjects	125	125
Eyes	125	125
Age (year)	69 (± 11)	74 (± 9)
Sex (F:M)	(73:52)	(67:58)
Laterality (R:L)	(62:63)	(63:62)

*F* female, *M* male, *R* right, L left

We hypothesized that when this postoperative posterior to preoperative anterior corneal curvature radii (PPPA) ratio differs from the PA ratio in virgin eyes, it will render the keratometer index invalid and cause a hyperopic shift after triple DMEK, similar to IOL power calculation in eyes after myopic photoablative refractive surgery [4].

The aim of this study was to assess the validity of conventional K for IOL power calculation in eyes with Fuch’s dystrophy undergoing triple DMEK using Scheimpflug imaging (Pentacam HR). Therefore, we compared the postoperative posterior to preoperative anterior corneal curvature radii (PPPA) ratio in eyes with Fuch’s dystrophy undergoing DMEK with the PA ratio calculated in healthy eyes.

Finally, to guide further IOL power calculation methods, the most appropriate ratio and keratometer index for the conversion of preoperative anterior corneal radius to postoperative posterior and total corneal powers was determined in a theoretical model.

## Methods

### Patients and examination

This retrospective study included patients with Fuchs endothelial corneal dystrophy (FECD), who underwent uncomplicated DMEK surgery in the Department of Ophthalmology of the University Hospital of Muenster and a control group consisting of eyes without corneal pathologies or prior ocular surgery. The study was approved by the local ethics committee and adhered to the tenets of the Declaration of Helsinki. Seventy-nine eyes included in the study have been part of a previous study by this group [14].

Eyes with a history of other corneal diseases, corneal infection or intraocular inflammation, trauma, corneal scars, contact lens wear 4 weeks before measurement, clinically significant graft detachment, or delayed corneal clearance were excluded.

All eyes underwent Scheimpflug corneal anterior segment tomography (Pentacam HR; Oculus, Wetzlar, Germany) in the same location under the same conditions with an expert examiner. Pentacam imaging in eyes with FECD undergoing DMEK was performed before surgery and after attaining refractive stability (minimum 3 months after surgery) [8].

Tomographic data was used in each eye to calculate a keratometer index and PA ratio before (*n*_c_^FECD^, *R*_PA_^FECD^) and after DMEK (*n*_c_^DMEK^, *R*_PA_^DMEK^) as well as a fictitious keratometer index (*n*_c_^FECD/DMEK^) based on PPPA ratio (*R*_PPPA_^FECD/DMEK^).

To assess the validity of conventional keratometry in eyes with Fuch’s dystrophy undergoing DMEK, the *R*_PPPA_^FECD/DMEK^ was compared to the PA ratio in the control group consisting of healthy corneas. It was hypothesized that when the PPPA ratio in eyes with Fuch’s dystrophy undergoing DMEK is significantly different to the PA ratio in virgin eyes, it will render conventional K invalid and the corneal power derived from it by this method.

Finally, in a theoretical model, the mean anterior corneal radius (*R*_A_) in each eye before DMEK was converted to postoperative posterior and total corneal powers, using the different calculated geometric mean PA ratios and keratometer indices. To determine the best fitting parameters, the predicted and measured powers were compared using Bland-Altman plots.

Calculation methods were as follows:

### Calculation of the posterior to anterior corneal curvature radii ratio

1 $$ {R}_{PA}=\frac{R_A}{R_P} $$

PA ratio was calculated for the FECD group (*R*_PA_^FECD^), DMEK group (*R*_PA_^DMEK^), and control group (*R*_PA_^Control^) using the geometric mean ratio between posterior (*R*_P_) and anterior (*R*_A_) corneal radii.

**Fig. 1:**
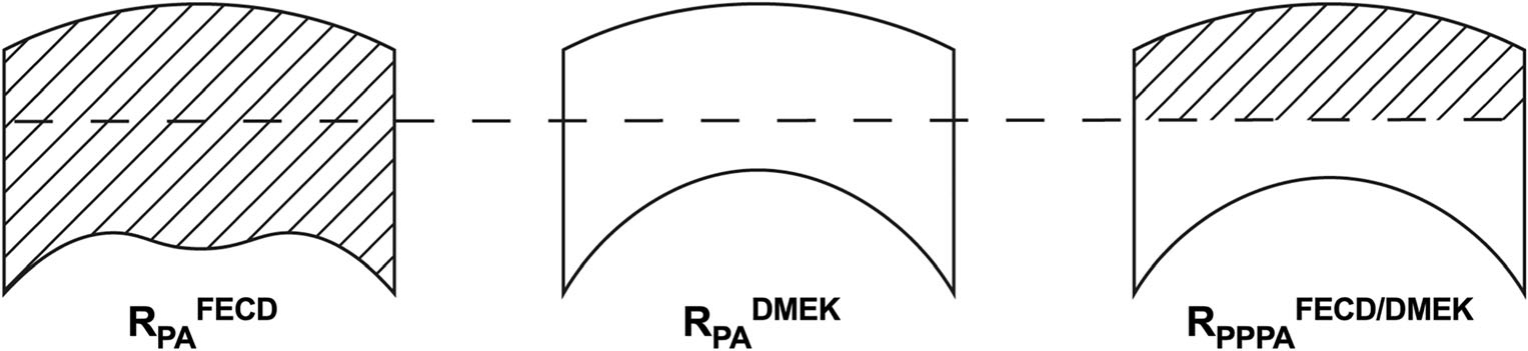
*R*_PPPA_^FECD/DMEK^ combines the posterior and anterior corneal curvature measurements when they are relevant for IOL power calculation: preoperative anterior corneal curvature when IOL power is calculated using conventional keratometry and postoperative posterior corneal curvature once stable refraction has been achieved

Furthermore, we calculated *R*_PPPA_^FECD/DMEK^ as the geometric mean ratio between preoperative anterior corneal radius (when conventional K is performed) and postoperative posterior corneal radius (once stable refraction is achieved) as shown in Fig. 1.

### Calculation of the keratometer indices

As for the PA ratios, the geometric mean keratometer indices (*n*_c_) were calculated for each group (*n*_c_^FECD^, *n*_c_^DMEK^, *n*_c_^Control^). In addition, a fictitious keratometer index was also calculated, combining preoperative (FECD) and postoperative (DMEK) measurements (*n*_c_^FECD/DMEK^):

2 $$ {D}_A=\frac{\left({n}_{Cornea}-1\right)\times 1000}{R_A} $$

3 $$ {D}_P=\frac{\left({n}_{aqueous}-{n}_{cornea}\right)\times 1000}{R_A\times {R}_{PA}} $$

4 $$ {D}_T=\frac{CCT}{n_{cornea}\times 1000}\times {D}_A\times {D}_P $$

5 $$ {D}_{Total}={D}_A+{D}_P-\frac{CCT}{n_{cornea}\times 1000}\times {D}_A\times {D}_P $$

6 $$ {n}_C=\frac{D_{Total}\times {R}_A}{1000}+1 $$

Formulas 2–6 were used to calculate *n*_c_^FECD^, *n*_c_^DMEK^, *n*_c_^Control^, and *n*_c_^FECD/DMEK^ using the anterior corneal curvature radius, central corneal thickness, and PA and PPPA ratios based on the thick lens formula. *D*_A_ is the dioptric power of the anterior corneal surface, *R*_A_ is the mean anterior corneal curvature radius, *D*_P_ is the dioptric power of the posterior corneal surface, *R*_PA_ is the individual posterior to anterior corneal curvature radii ratio, *n*_cornea_ is the refractive index of the cornea (1.376), *n*_aqueous_ is the refractive index of aqueous (1.336), and *D*_Total_ is the dioptric power of the cornea, while CCT is the mean central corneal thickness. For the calculation of *n*_c_^FECD/DMEK^, *R*_PA_ is replaced by the *R*_PPPA_ in Formula 3.

### Predicting postoperative posterior and total corneal powers

Different geometric mean ratios and keratometer indices were used to convert mean anterior corneal radius (*R*_A_) in each eye before DMEK to postoperative posterior (Formula 7) and total corneal power (Formula 9) respectively.

Measured anterior corneal radius (*R*_A_) of each eye before DMEK was converted to postoperative posterior corneal power (*D*_P_^Pred.^) using different calculated geometric mean ratios (*R*_PA_ = *R*_PA_^FECD^, *R*_PA_^Control^, *R*_PPPA_^FECD/DMEK^) (Formula 7). Results were compared with the corresponding measured posterior corneal power after DMEK (*D*_P_^DMEK^) (Formula 8).

7 $$ {D}_{{\mathrm{P}}^{\mathrm{P}\mathrm{red}.}}=\frac{\left({n}_{\mathrm{aqueous}}-{n}_{\mathrm{cornea}}\right)\times 1000}{{\mathrm{R}}_{\mathrm{A}}\ \mathrm{x}\ {\mathrm{R}}_{\mathrm{P}\mathrm{A}}} $$

8 $$ {\Delta  D}_{\mathrm{P}}={D}_{{\mathrm{P}}^{\mathrm{DMEK}}}-{D}_{{\mathrm{P}}^{\mathrm{P}\mathrm{red}.}.} $$

where *D*_P_^Predicted^ (*D*_P_^Pred.^) is the mean predicted postoperative posterior corneal power, *n*_cornea_ is the refractive index of the cornea (1.376), *n*_aqueous_ is the refractive index of aqueous (1.336), *R*_PA_ is the geometric mean posterior to anterior corneal curvature radii ratio for the given groups (*R*_PA_^FECD^ = 0.900; *R*_PA_^Control^ = 0.822; *R*_PPPA_^FECD/DMEK^ = 0.806), and *r*_A_ is the preoperative anterior corneal radius of a single Pentacam. *D*_P_^DMEK^ is the measured postoperative posterior corneal power (Formula 3) and *∆D*_P_ is the difference between predicted and measured postoperative posterior corneal power. Three different *∆D*_P_ were calculated for each eye, based on the mentioned geometric mean PA ratios.

Measured anterior corneal radius (*R*_A_) of each eye before DMEK was converted to postoperative total corneal power (*D*_T_^Pred.^) using the different calculated geometric mean keratometer indices (*n*_c_^FECD^, n_c_^Control^, *n*_c_^FECD/DMEK^) (Formula 9). Results were compared with the corresponding total corneal power measured after DMEK (*D*_Total_^DMEK^) (Formula 10).

9 $$ {D}_{{\mathrm{T}}^{\mathrm{Pred}.}}=\frac{n_{\mathrm{c}}}{R_{\mathrm{A}}}-1 $$

10 $$ {\Delta  D}_{\mathrm{T}\mathrm{otal}}={D}_{{\mathrm{T}\mathrm{otal}}^{\mathrm{DMEK}}}-{D}_{{\mathrm{T}}^{\mathrm{Pred}.}} $$

where *D*_T_^Predicted^ (*D*_T_^Pred.^) is the mean predicted postoperative total corneal power, *n*_c_ is the geometric mean fictitious keratometer index for the given groups (*n*_c_^FECD^ = 1.3319, *n*_c_^Control^ = 1.3283, *n*_c_^FECD/DMEK^ = 1.3273), and *R*_A_ is the preoperative anterior corneal radius of a single Pentacam. *D*_T_^DMEK^ is the measured postoperative total corneal power (Formula 5) and *∆D*_Total_ is the difference between predicted and measured postoperative total corneal power. Three different *∆D*_Total_ were calculated for each eye, based on the mentioned geometric mean keratometer indices.

**Fig. 2:**
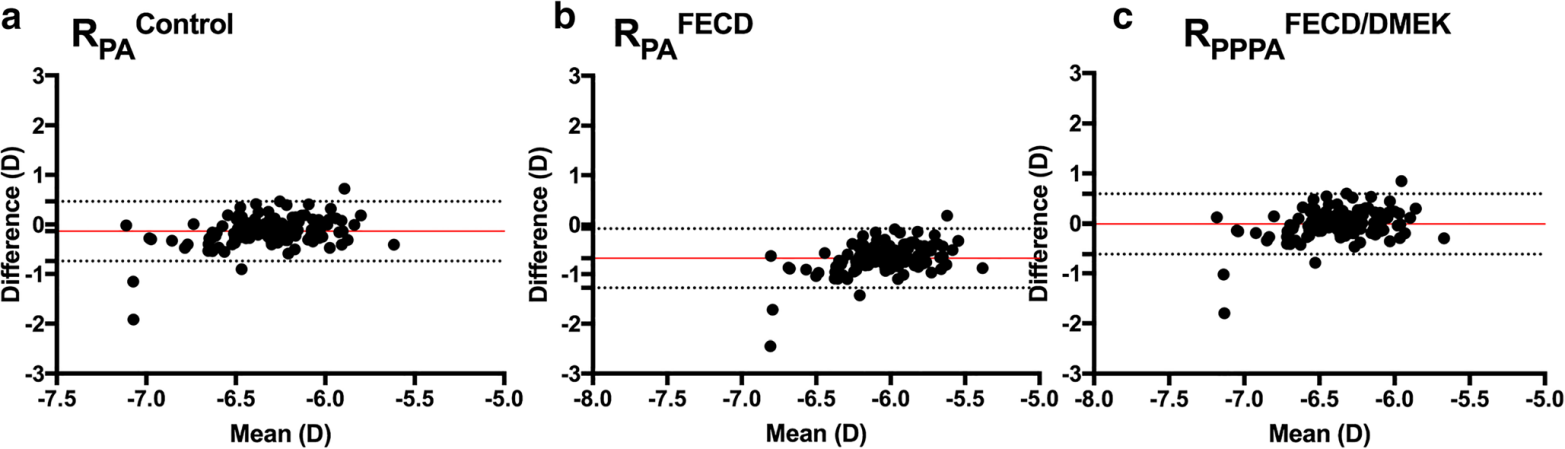
Bland-Altman plot comparing the measured and predicted postoperative posterior corneal power calculated with different PA ratios and the PPPA ratios respectively. Mean differences are represented by solid lines, while 95% limits of agreement (LoA) are represented by dotted lines. **a** Using *R*_PA_^Control^ 0.822, **b** using *R*_PA_^FECD^ 0.900, and **c** using *R*_PPPA_^FECD/DMEK^ 0.806

**Fig. 3:**
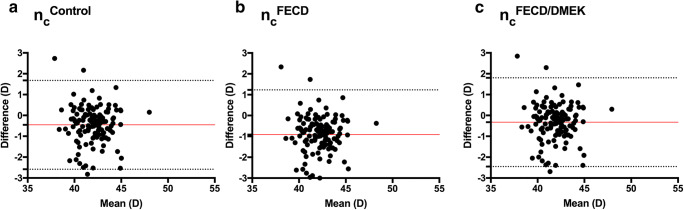
Bland-Altman plot comparing the measured and predicted postoperative corneal power calculated with different keratometer indices. Mean differences are represented by solid lines, while 95% limits of agreement (LoA) are represented by dotted lines. **a** Using *n*_c_^Control^ = 1.3283, **b** using *n*_c_^FECD^ = 1.3319, and **c** using *n*_c_^FECD/DMEK^ = 1.3273

Furthermore, we compared predicted and measured postoperative posterior and total corneal power using Bland-Altman plots (Figs. 2 and 3).

### Surgical procedure

At two and ten o’clock positions, two paracenteses were performed. After filling the anterior chamber with air, a 9-mm descemetorhexis was performed using a Sinskey hook.

A 2.8-mm incision was made at the limbus for the insertion of the graft. The 8.75-–9.00-mm donor Descemet roll was stained with a 0.06% trypan blue solution (Vision Blue, D.O.R.C. International) and sucked in to a glass injector (DMEK-Inserter, Geuder, Germany) for injection into the anterior chamber.

The graft was oriented in the center with endothelial side down by indirect manipulation with air and BSS. To position the graft onto the recipient posterior stroma, an air bubble was injected underneath the graft. After surgery, patients were asked to maintain a supine position for at least 4 h.

### Statistics

Microsoft Excel 2010 was used for data management. Statistical analyses were performed with IBM SPSS® Statistics 22 for Windows (IBM Corporation, Somers, NY, USA).

Data were reported as mean ± standard deviation (median [25, 75 percentiles]).

The normality of the data distribution was tested using the Kolmogorov–Smirnov test.

A normal distribution was not found for *R*_PA_, *R*_P_, *D*_P_, CCT, or the keratometer index in eyes with FECD; nor was the distribution normal for *D*_P_ and the keratometer index in eyes after DMEK surgery or for *R*_A_ in the control group.

Depending on the normality distribution, postoperative data were compared to baseline using the paired *t* test or two-sided Wilcoxon signed-rank test, while the differences in relation to control group data were determined using an unpaired *t* test or Man-Whitney *U* test.

Bland-Altman plots were used to evaluate the agreement between measured and predicted postoperative corneal parameters (*∆D*_P_ and *∆D*_Total_). Bland-Altman plots allow one to determine whether there are systematic differences between measured and calculated parameters. The mean difference is the estimated bias, and the standard deviation of the difference measures the random fluctuations around this mean. The level of statistical significance was set at *P* ≤ 0.05. A post hoc power analysis (G*power, version 3.1) was conducted to determine the study power.

## Results

A total of 125 eyes of 125 patients who underwent DMEK or triple DMEK and 125 eyes of 125 patients without corneal pathologies were included in this retrospective study. Patients’ characteristics are summarized in Table 1. Postoperative changes relative to baseline are shown in Table 2.

**Table 2:** Comparison of preoperative and postoperative tomographic data

	FECD	DMEK		
	Mean ± SD; *	Mean ± SD; *	Change (*∆*) ± SD	*P* value
*R*_A_ (mm)	7.80 ± 0.33;(7.70[7.57, 8.01])	7.84 ± 0.31(7.83[7.63, 8.08])	0.05 ± 0.19	**= 0.001**^**1**^
*D*_A_ (D)	48.30 ± 2.04; (48.39[46.94, 49.67])	47.98 ± 1.89; (48.02[46.53, 49.28])	− 0.31 ± 1.10	**< 0.001**^**1**^
*R*_P_ (mm)	6.98 ± 0.80; (6.82[6.48, 7.25])	6.28 ± 0.35; (6.29[6.09, 6.53])	− 0.70 ± 0.81	**< 0.001**^**1**^
*D*_P_ (D)	−5.80 ± 0.62;(−5.87[−6.17, −5.51])	−6.39 ± 0.37;(−6.36[−6.57, −6.12])	− 0.59 ± 0.66	**< 0.001**^**1**^
CCT (μm)	660 ± 106;(640[605, 690])	530 ± 50;(527[497, 560])	− 130 ± 113	**< 0.001**^**1**^
*R*_PA_	0.90 ± 0.10(0.87[0.84, 0.93)]	0.801 ± 0.04;(0.80[0.78, 0.83])	− 0.1 ± 0.11	**< 0.001**^**1**^
*D*_Total_ (D)	42.63 ± 2.04;(42.77[41.28, 43.91])	41.72 ± 1.72; (41.78[40.25, 42.75])	− 0.92 ± 1.47	**< 0.001**^**1**^
*n*_c_	1.3319 ± 0.005;(1.3331[1.330, 1.334])	1.3270 ± 0.002;(1.3270[1.326, 1.328])	− 0.005 ± 0.006	**< 0.001**^**1**^

significant *P* values: bold; *SD* standard deviation ^*^ = (median [25, 75 percentiles]); 1 = Wilcoxon, *CCT* central corneal thickness, *R*_*A*_ average reading of the anterior corneal curvature, *R*_*P*_ average reading of the posterior corneal curvature, *D*_*A*_ anterior corneal power; *D*_*P*_ posterior corneal power, *D*_*Total*_ total corneal power, *R*_*PA*_ posterior to anterior corneal curvature radii ratio, *n*_*c*_ fictitious keratometer index

Eyes after DMEK surgery showed a statistically significant thinner central corneal thickness, steeper posterior corneal radius, and lower PA ratio when compared to eyes without corneal pathologies (Table 3).

**Table 3:** Comparison of measured control group data with FECD, DMEK, and FECD/DMEK group data

	Control Mean ± SD;*	FECD		DMEK		FECD/DMEK	
		Mean ± SD;*	***P***	Mean ± SD;*	***P***	Mean ± SD;*	***P***
*R*_A_ (mm)	7.83 ± 0.28; (7.80[7.64, 7.98])	7.80 ± 0.33;(7.70[7.57, 8.01])	> 0.05^4^	7.84 ± 0.31(7.83[7.63, 8.08])	> 0.05^4^		
*D*_A_ (D)	48.09 ± 1.71; (48.21[47.12, 49.21])	48.30 ± 2.04; (48.39[46.94, 49.67])	> 0.05^3^	47.98 ± 1.89; (48.02[46.53, 49.28])	> 0.05^3^		
*R*_P_ (mm)	6.44 ± 0.27; (6.43[6.27, 6.57])	6.98 ± 0.80; (6.82[6.48, 7.25])	**< 0.001**^**4**^	6.28 ± 0.35; (6.29[6.09, 6.53])	**< 0.001**^**3**^		
*D*_P_ (D)	− 6.23 ± 0.26;(−6.22[− 6.39, −6.09])	− 5.80 ± 0.62;(− 5.87[− 6.17, − 5.51])	**< 0.001**^**4**^	− 6.39 ± 0.37;(− 6.36[− 6.57, − 6.12])	**< 0.001**^**4**^		
CCT (μm)	553 ± 36; (556[530, 578])	660 ± 106;(640[605, 690])	**< 0.001**^**4**^	530 ± 50;(527[497, 560])	**< 0.001**^**3**^		
*R*_PA_	0.822 ± 0,02; (0.82[0.81, 0.83])	0.90 ± 0.10(0.87[0.84, 0.93)]	**< 0.001**^**4**^	0.801 ± 0.04;(0.80[0.78, 0.83])	**< 0.001**^**3**^	*0.806 ± 0.03; (0.80[0.79, 0.83])	**= 0.001**^**3**^
*D*_Total_ (D)	41.98 ± 1,50; (42.14[41.09, 42.94])	42.63 ± 2.04;(42.77[41.28, 43.91])	**< 0.001**^**3**^	41.72 ± 1.72; (41.78[40.25, 42.75])	> 0.05^3^	42.03 ± 1.86; (42.20[40.63, 43.24])	> 0.05^3^
*n*_c_	1.3283 ± 0.001; (1.328[1.328, 1.329])	1.3319 ± 0.005;(1.3331[1.330, 1.334])	< **0.001**^**4**^	1.3270 ± 0.002;(1.3270[1.326, 1.328])	**< 0.001**^**4**^	1.3273 ± 0.002; (1.3272[1.326, 1.329])	**< 0.001**^**4**^

Significant *P* values: bold; *SD* standard deviation * = (median [25, 75 percentile]); 3 = unpaired t-test; 4 = Man-Whitney *U* test; *CCT* central corneal thickness, *R*_*A*_ average reading of the anterior corneal curvature, *R*_*P*_ average reading of the posterior corneal curvature, *D*_*A*_ anterior corneal power, *D*_*P*_ posterior corneal power, *D*_*Total*_ total corneal power, *R*_*PA*_ *order PA ratio* posterior to anterior corneal curvature radii ratio, *n*_*c*_ fictitious keratometer index; * = calculated using the R_PPPA_

The PPPA ratio and keratometer index determined in eyes with Fuch’s dystrophy undergoing DMEK was significantly different (*P* < 0.001) to the keratometer index and PA ratio in eyes without corneal pathologies (Table 3). In a post hoc power analysis, the calculated effect size was 0.62 (ratios) and 0.63 (keratometer indices). With an alpha of 0.05, this led to a power of 0.99 in both cases.

Using the geometric mean *R*_PA_^Control^ (0.822) and *n*_c_^Control^ (1.3283) to convert the anterior corneal radius before DMEK to power leads to significant (*P* < 0.001) underestimation of the negative postoperative posterior (*∆D*_P_ = − 0.14D ± 0.30) and thus overestimation of the total corneal power (*∆D*_Total_ = −0.45D ± 1.08) (Table 4).

**Table 4:** Comparison of predicted and measured postoperative posterior (*∆D*_P_) and total corneal powers (*∆D*_Total_) using different ratios and fictitious keratometer indices

	Control *R*_PA_ = 0.822 *n*_c_ = 1.3283		FECD *R*_PA_ = 0.900 *n*_c_ = 1.3319		FECD/DMEK *R*_PPPA_ = 0.806 *n*_c_ = 1.3273	
	Mean ± SD	*P* value	Mean ± SD	*P* value	Mean ± SD	*P* value
*∆D*_P_	− 0.14 ± 0.30	**< 0.001**	− 0.68 ± 0.30	**< 0.001**	− 0.01 ± 0.30	> 0.05
*∆D*_Total_	− 0.45 ± 1.08	**< 0.001**	− 0.92 ± 1.08	**< 0.001**	− 0.32 ± 1.08	**< 0.001**

Significant *P* values: bold; *SD* standard deviation, *∆D*_*Total*_ difference between predicted and measured total corneal power, *∆D*_*P*_ difference between predicted and measured postoperative posterior corneal power, *n*_*c*_ fictitious keratometer index, *R*_*PA*_ posterior to anterior corneal curvature radii ratio, *R*_*PPPA*_ postoperative posterior to preoperative anterior corneal curvature radii ratio, *Contro*l control group data; *FECD* preoperative data of eyes with FECD, *FECD/DMEK* postoperative posterior and preoperative anterior corneal curvature data of eyes with FECD undergoing DMEK surgery

The lowest prediction errors were found using the mean fictitious *R*_PA_^FECD/DMEK^ (0.806) and *n*_c_^FECD/DMEK^ (1.3273) to predict the postoperative posterior (*∆D*_P_ = − 0.01D ± 0.30) and total corneal powers (*∆D*_Total_ = − 0.32D ± 1.08) (Table 4).

Figures 2 and 3 show the Bland-Altman plots comparing predicted and measured postoperative posterior and total corneal powers (Figs. 2 and 3).

## Discussion

Ours is the first study to demonstrate that using conventional keratometry to estimate postoperative corneal refractive power in patients with FECD undergoing DMEK surgery, leads to an overestimation of corneal power. Through the introduction of a fictitious PPPA ratio and keratometer index, we have found a promising way to adjust conventional keratometry and provide the necessary correction.

The 4 potential sources of error in IOL calculation are corneal curvature measurement, axial length measurement, effective lens position estimation, and the IOL calculation formula [15–17].

This study focused on corneal power measurement that accounts for approximately two-thirds of the total dioptric power of the eye [17]. If the calculation of corneal power is inaccurate, it will have profound consequences on subsequent steps in the calculation of IOL power [18–21].

Optical biometers such as the IOL Master 500 (Carl Zeiss Meditec, Jena, Germany) do not measure the posterior cornea directly, but instead account for it using a fictitious refractive index of the cornea, under the assumption that the posterior to anterior corneal curvature radii ratio is constant in all eyes [1]. In this study, the posterior corneal power changed significantly after DMEK surgery, a finding in line with all related studies presented in the literature [5–12] (Table 2).

From the combined information presented above, we conclude that the relationship between the postoperative posterior corneal radius—once stable refraction is achieved—and to the preoperative anterior corneal curvature—when conventional K is performed—is decisive for IOL power calculation (IOL Master 500) in eyes undergoing DMEK surgery (Fig. 1).

Furthermore, we have demonstrated that this ratio is significantly different to the PA ratio in healthy eyes, so that the keratometric index and the corneal power derived from it by this method will become invalid [18, 19].

This is similar to the situation with altered PA ratio after myopic laser vision correction (e.g., LASIK, PRK), where conventional K overestimates corneal power, which leads to a biased IOL calculation and hyperopic outcome after cataract surgery [4, 19, 22–25].

To counteract this, modern optical biometers (e.g., IOLMaster 700) are capable of measuring both anterior and posterior corneal curvatures, to assess the total keratometry (TK). With the use of innovative IOL calculation methods, such as the Haigis-TK or Barret True-K formula, this enables a more precise outcome after cataract surgery with previous LASIK or PRK [4, 26, 27].

In this context, it is important to mention that in eyes with Fuch’s dystrophy undergoing DMEK, the posterior corneal curvature changes significantly after the performed surgery. Therefore, considering the preoperative posterior corneal curvature in IOL power calculation would lead to an overestimation of the postoperative corneal power (Table 4).

In our study, DMEK leads to a significant thinner central corneal thickness, steeper posterior corneal curvature, and thereby lower PPPA and postoperative PA ratios when compared to healthy eyes (Table 3). Similar results are presented in the literature. Arnalich-Montiel et al. showed a thinner cornea with a steeper pachymetric progression from the thinnest point to the periphery in eyes after DMEK compared to normal corneas [28].

This difference could be simply explained by the fact that DMEK surgery does not replace the entire endothelium and Descemet membrane (DMEK roll size usually lies between 8.0 and 9.25 mm).

However, a standard IOL power calculation using conventional K is inaccurate in the case of triple DMEK and will lead to a significant underestimation of posterior negative corneal power and hence overestimation of total corneal power (Table 4).

In the clinical practice, DMEK surgeons have noticed this problem and aim to achieve a more myopic postoperative outcome by choosing a refractive target of − 0.5 to − 1D to compensate for this error [8]. In the future, IOL power calculation could be optimized for eyes with FECD undergoing triple DMEK surgery by using adjusted conventional keratometry to improve the predictability of IOL power calculation and account for the postoperative hyperopic shift [18, 21, 29–31].

In comparison to that, a higher postoperative hyperopic error has been reported in eyes undergoing Descemet stripping endothelial keratoplasty (DSEK) combined with cataract surgery [32]. This could also be explained by a non-physiologic postoperative posterior corneal curvature that is also overestimated by conventional K. Goldich et al. found a significantly steeper posterior corneal radius after DSEK compared to eyes undergoing DMEK. In contrast, there was no difference when comparing the anterior corneal curvature preoperative and postoperative in between the groups [33].

Using the PPPA ratio presented in this study (0.806) enhances the accuracy of the refractive prediction of postoperative posterior corneal power in comparison with the PA ratio of eyes with FECD (0.900) or healthy corneas (0.822) (Fig. 2).

Compared to that, using the fictitious keratometer index (1.3723) to preoperatively predict postoperative total corneal power leads to a minimal additional overestimation of that power on average. This is caused by a significant postoperative flattening of the anterior corneal curvature in the present data (Fig. 3).

This is similar to the results of our previous study, where we noted a more strongly correlation between preoperative corneal parameters (e.g., PA ratio, Asph. Q) and the change in refractive power of the posterior corneal surface compared to the change in total corneal power [14].

Regarding the change of the anterior corneal curvature after DMEK, different results are presented in the literature. Kwon et al. found no significant alteration of simulated keratometry after surgery [10], whereas van Dijk et al. showed an ongoing significant change in mean anterior corneal curvature [11].

The variation between predicted and measured postoperative total corneal power (± 1.08D) indicates that changes of anterior corneal curvature could explain the problem of varying postoperative refractive outcomes after triple DMEK that go from myopia to hyperopia.

However, a keratometer index cannot account for these changes [34] and the prediction of the individual refractive outcome in eyes with Fuch’s dystrophy prior to Triple DMEK will remain a challenging task for surgeons.

This pilot study was limited by a relatively small sample size and the retrospective design. This may have affected the results to a minimal extent. Nevertheless, a high power was achieved in the post hoc power analysis.

Moreover, the results presented in this monocenter study may be skewed by specific surgical details (incision at 12 h and 8.75-–9.00-mm transplant); this ratio and keratometer index could easily be evaluated for the different surgeons or different centers. In any case, multicenter studies with a higher number of patients are needed.

In conclusion, the postoperative posterior to preoperative anterior corneal curvature radii ratio is the decisive ratio for IOL power calculation in eyes undergoing triple DMEK and differs significantly from the PA ratio in healthy eyes. Conventional K for IOL power calculation in eyes with FECD undergoing triple DMEK surgery should therefore be corrected to avoid overestimation of postoperative corneal power and to minimize the risk of a hyperopic shift. The newly derived PPPA ratio and corresponding fictitious keratometer index *n*_c_^FECD/DMEK^ represent a promising corrective tool. Further studies with innovative IOL power calculation methods are needed to evaluate the utility of this parameter in patients undergoing triple DMEK.
